# Visual Field Loss and Self-Reported Driving Restriction in Glaucoma

**DOI:** 10.3390/vision10020025

**Published:** 2026-04-29

**Authors:** Mladena Radeva, Preslava Encheva, Elitsa Hristova, Daliya Stefanova, Igor Resnick, Zornitsa Zlatarova

**Affiliations:** 1 University Specialized Eye Hospital, Doyran 15 Str., 9002 Varna, Bulgaria; 2 Department of Ophthalmology and Visual Sciences, Medical University of Varna, 9002 Varna, Bulgaria; 3 Department of Optometry and Occupational Diseases, Medical University of Varna, 9002 Varna, Bulgaria; 4 Department of Medical Genetics, Medical University of Varna, 9002 Varna, Bulgaria

**Keywords:** glaucoma, visual field loss, driving restriction, self-regulation, perimetry, mean deviation, visual field index, quality of life

## Abstract

**Background:** To evaluate the association between glaucomatous visual field loss and self-reported driving limitation, and to explore potential threshold ranges of visual field loss associated with an increased likelihood of driving restriction. **Methods:** In this cross-sectional study, 100 patients with primary open-angle glaucoma underwent standard automated perimetry. Visual function was assessed using Mean Deviation (MD) and Visual Field Index (VFI) from the better eye. Driving status, driving limitation, and self-reported driving difficulties were assessed using a structured questionnaire. Multivariable logistic regression was performed to determine independent associations between visual field parameters and driving limitation, adjusting for age, sex, cataract status, and systemic comorbidities. Because MD and VFI are closely related indices of visual field loss, separate multivariable models were constructed for each parameter. Receiver operating characteristic (ROC) analysis was used to explore threshold values associated with driving limitation. **Results:** Driving limitation increased progressively with worsening functional severity, affecting 17% of participants with preserved function, 48% of those with borderline impairment, and 72% of those with definite impairment (*p* < 0.001). Reduced VFI was independently associated with driving limitation (OR = 0.972, 95% CI: 0.948–0.996; *p* = 0.021). In a separate model, more negative MD was also independently associated with driving limitation (OR = 0.924, 95% CI: 0.875–0.976; *p* = 0.004). Male sex was associated with a lower likelihood of driving limitation. ROC analysis identified threshold values of VFI ≤ 71% (AUC = 0.663) and MD ≤ −13.36 dB (AUC = 0.650), both characterized by high specificity but limited sensitivity. Participants who had ceased driving demonstrated worse visual field indices than active drivers, whereas never-drivers showed no consistent association with visual field loss. **Conclusions:** Glaucomatous visual field loss was significantly associated with self-reported driving limitation and behavioural self-regulation. Objective perimetric parameters, particularly VFI and MD in the better eye, may help identify patients more likely to report driving difficulties. The reported threshold values should be interpreted as exploratory reference points rather than clinically actionable criteria and require further validation before clinical application.

## 1. Introduction

Glaucoma is a leading cause of irreversible visual impairment worldwide and is characterized by progressive loss of retinal ganglion cells and their axons, resulting in corresponding visual field defects [1,2]. Although central visual acuity may remain relatively preserved until advanced stages, peripheral visual field loss can substantially impair complex daily activities that depend on spatial awareness, hazard detection, divided attention, and rapid visual processing. Among these, driving is one of the most functionally demanding and socially significant tasks [3,4,5].

Driving is closely linked to personal independence, employment, social participation, and overall quality of life [4,5]. Accordingly, assessment of driving-related functional difficulties in patients with glaucoma represents both a clinical and public health challenge. Physicians must balance patient autonomy with road safety, often in the absence of clearly defined functional thresholds that translate ophthalmic findings into meaningful real-world limitations [6].

Historically, assessment of driving eligibility has relied predominantly on visual acuity criteria. However, increasing evidence suggests that visual field function may be more relevant than acuity alone for maintaining unrestricted driving activity, particularly in glaucoma [6,7]. Patients with glaucomatous field loss may experience difficulty detecting peripheral hazards, responding to dynamic traffic situations, or driving under challenging conditions such as low illumination [6,7,8,9]. Nevertheless, the relationship between measured visual field damage and self-reported driving behaviour remains incompletely understood.

Driving-related outcomes in visually impaired individuals include several related but distinct constructs. Driving limitation refers to reduced or modified driving activity, such as avoiding night driving or decreasing driving frequency [10]. Driving cessation indicates complete discontinuation of driving. Self-regulation describes adaptive behavioural changes adopted in response to perceived or actual functional difficulty. These outcomes may overlap, but they are not equivalent to objectively measured driving performance, collision risk, or formal fitness-to-drive status.

Among commonly used perimetric indices, Mean Deviation (MD) reflects the overall reduction in visual field sensitivity relative to age-matched normative values, whereas the Visual Field Index (VFI) estimates remaining visual function as a percentage [11]. Both are widely used global markers of glaucomatous damage and may be clinically relevant for driving-related functioning [12]. However, some patients continue to drive despite measurable visual field loss, whereas others voluntarily restrict or cease driving. This variability suggests that structural or perimetric severity alone may not fully explain behavioural adaptation.

At the same time, self-perceived driving ability or confidence may not reliably reflect objective impairment, particularly when central acuity remains preserved. Identifying clinically meaningful thresholds of visual field loss associated with behavioural change may therefore improve patient counselling and support more individualized clinical assessment.

The primary aim of this study was to evaluate the association between visual field impairment and self-reported driving limitation in patients with glaucoma. The secondary aim was to explore potential threshold ranges of visual field loss associated with an increased likelihood of driving restriction. Establishing such preliminary reference values may support future research and assist clinicians in contextual discussions with patients, while requiring prospective validation before clinical implementation.

## 2. Materials and Methods

### 2.1. Study Design and Participants

This cross-sectional clinical study included 100 consecutive patients with a confirmed diagnosis of glaucoma who underwent standard automated perimetry as part of routine ophthalmic evaluation. All participants completed a structured questionnaire assessing driving behaviour, perceived visual difficulties during driving, and relevant clinical history.

Participants were recruited from a tertiary ophthalmology centre. Inclusion criteria were: (1) confirmed glaucoma diagnosis; (2) availability of reliable visual field testing; and (3) completed questionnaire data. Exclusion criteria included neurological disorders affecting the visual field and ocular diseases unrelated to glaucoma that could substantially impair visual function, except for cataract, which was recorded separately as a potential confounding factor.

The study adhered to the tenets of the Declaration of Helsinki and was approved by the Ethics Committee of the Medical University of Varna (Protocol No. 22/20 November 2025). Written informed consent was obtained from all participants.

### 2.2. Visual Field Assessment

Standard automated perimetry was performed using the Humphrey Field Analyzer 3 (Carl Zeiss Meditec, Dublin, CA, USA) with the 24-2 SITA Standard protocol [13]. 

Only reliable tests were included, defined as fixation losses ≤ 20%, false-positive responses ≤ 15%, and false-negative responses ≤ 15%.

The following global visual field indices were recorded for each eye:

- Mean Deviation (MD, dB)
- Visual Field Index (VFI, %)
- Pattern Standard Deviation (PSD, dB)

For the primary analyses, values from the better eye were used, defined as the eye with higher VFI and less negative MD. This approach was selected as a pragmatic approximation of functional vision commonly used in clinical studies, although it does not fully account for binocular visual field integration.

Inter-eye asymmetry was calculated as the absolute interocular difference between eyes for MD and VFI:

- |MD OD − OS|
- |VFI OD − OS|

For descriptive purposes, visual field severity was categorized according to better-eye MD as follows:

- Mild defect: MD > −6 dB
- Moderate defect: MD between −6 and −12 dB
- Severe defect: MD < −12 dB

In addition to quantitative indices, participants were clinically stratified into three functional severity groups based on expert interpretation of visual field data: preserved function, borderline impairment, and definite functional impairment. This stratification was used for descriptive and trend analyses only and was not entered as a predictor in regression models. Classification was performed by an experienced clinician, was not formally masked, and interobserver agreement was not assessed.

Because MD and VFI are global indices, they do not provide detailed information regarding the spatial location of defects (e.g., central or paracentral loss). Localized defect analysis was not performed.

### 2.3. Assessment of Driving Behaviour and Functional Outcomes

Driving behaviour was assessed using a structured, study-specific questionnaire designed to capture clinically relevant aspects of functional driving behaviour in glaucoma.

The questionnaire included closed-ended items addressing:

- current driving status
- driving frequency
- avoidance of night driving
- driving cessation
- self-reported visual difficulties during driving
- history of driving incidents

Although the questionnaire was not formally validated, all participants completed the same standardized instrument under identical conditions.

Driving limitation was defined as a composite outcome including reduced driving frequency, avoidance of specific driving conditions (particularly night driving), or complete cessation of driving. This endpoint was chosen to capture the spectrum of functional driving restriction and behavioural self-regulation.

In addition to the composite endpoint, individual questionnaire items were analysed separately.

Participants were further categorized as:

- active drivers
- former drivers (ceased driving)
- never drivers

Where applicable, reasons for cessation were recorded.

Subjective visual difficulties were categorized into predefined groups:

- peripheral vision problems
- night vision difficulty
- general visual uncertainty during driving
- no visual complaints

Behavioural self-regulation was defined as reduced driving frequency, avoidance of night driving, or complete cessation of driving.

### 2.4. Clinical and Demographic Variables

The following additional variables were recorded:

- age
- sex
- cataract status
- presence of systemic comorbidities

Cataract was considered a potential confounder because of its known influence on functional vision, particularly under low illumination, glare, and night driving conditions.

For primary analyses, cataract status was entered as a binary covariate (present/absent) in multivariable models.

### 2.5. Statistical Analysis

Data distribution was assessed using the Shapiro–Wilk test. Continuous variables are presented as mean ± standard deviation or median (interquartile range), as appropriate.

Between-group comparisons were performed using the Mann–Whitney U test for two-group analyses and the Kruskal–Wallis test for comparisons involving more than two groups. Associations between continuous variables were assessed using Spearman’s rank correlation.

Binary logistic regression analysis was performed to identify independent predictors of driving limitation. Because MD and VFI represent closely related global measures of visual field loss, separate multivariable logistic regression models were constructed for each parameter to minimize collinearity and improve interpretability. Both models were adjusted for age, sex, cataract, and systemic comorbidities.

Receiver operating characteristic (ROC) curve analysis was used to evaluate the ability of visual field parameters to discriminate driving limitation. Optimal cut-off values were determined using the Youden index, defined as the maximum value of sensitivity + specificity − 1.

Given the exploratory nature of the study and the number of outcomes assessed, no formal adjustment for multiple comparisons was applied. Results were interpreted with appropriate caution. Statistical significance was set at *p* < 0.05. All statistical analyses were performed using SPSS version 23.0.

## 3. Results

### 3.1. Study Population

The study included 100 patients with glaucoma (mean age 63.8 ± 14.4 years; 52% male). Cataract was present in 68% of participants, and systemic comorbidities were reported in 76%.

Based on predefined functional severity stratification, participants were categorized into three groups: preserved function (*n* = 54), borderline impairment (*n* = 21), and definite functional impairment (*n* = 25).

This stratification revealed a progressive gradient in functional visual loss and driving behaviour, supporting a dose–response relationship between worsening visual field status and driving limitation (Table 1).

### 3.2. Functional Severity Stratification and Visual Field Indices

Visual field indices worsened progressively across the three functional severity groups. Median better-eye VFI declined from 97 in the preserved group to 84 in the borderline group and 62 in the definite impairment group (*p* < 0.001). Median MD showed corresponding deterioration from −2.9 dB to −7.6 dB and −14.8 dB, respectively (*p* < 0.001).

These findings support the validity of the clinical stratification in reflecting increasing functional visual impairment (Table 1).

### 3.3. Functional Severity and Driving Behaviour

Driving limitation increased progressively with worsening functional severity, affecting 17% of participants with preserved function, 48% of those with borderline impairment, and 72% of those with definite impairment (*p* < 0.001).

Participants in the definite impairment group were significantly more likely to restrict driving, avoid night driving, and cease driving altogether. Non-driving status was also more frequent with increasing severity (9%, 24%, and 28%, respectively; *p* < 0.001).

Subjective driving complaints increased across severity groups (28%, 52%, and 68%, respectively; *p* = 0.01).

### 3.4. Visual Field Parameters and Driving Limitation

Across the full cohort, participants with driving limitation had significantly worse visual field function than those without limitation. Better-eye VFI was significantly lower, and better-eye MD was significantly more negative in participants with driving limitation (both *p* < 0.01). PSD showed a non-significant trend toward higher values in the driving limitation group.

These findings were consistent with the graded pattern observed across the predefined functional severity groups.

### 3.5. Predictors of Driving Limitation

In multivariable logistic regression adjusted for age, sex, cataract, and systemic comorbidities, reduced VFI remained independently associated with driving limitation in Model 1 (OR = 0.972, 95% CI: 0.948–0.996; *p* = 0.021).

In a separate model, more negative MD was also independently associated with driving limitation in Model 2 (OR = 0.924, 95% CI: 0.875–0.976; *p* = 0.004).

Male sex was associated with a lower likelihood of driving limitation, whereas age, cataract, and systemic comorbidities were not significant predictors in either model (Table 2).

### 3.6. Functional Thresholds for Driving Limitation

Receiver operating characteristic analysis was performed to assess the ability of visual field indices to discriminate driving limitation.

For VFI, the area under the curve (AUC) was 0.663 (95% CI: 0.548–0.774). The threshold identified by the Youden index was ≤71%, corresponding to a sensitivity of 46.2% and a specificity of 88.5%.

For MD, the AUC was 0.650 (95% CI: 0.528–0.764). The threshold identified by the Youden index was ≤−13.36 dB, with a sensitivity of 43.6% and a specificity of 90.0%.

**Figure 1:**
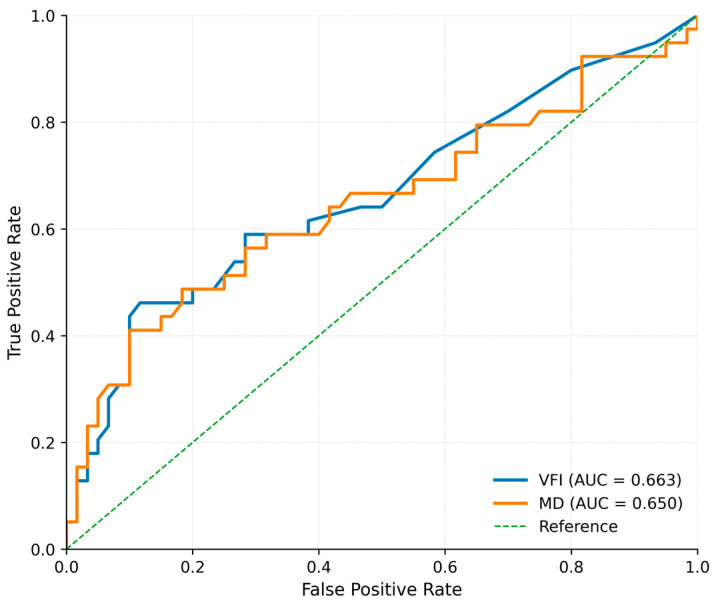
Receiver operating characteristic (ROC) curves for VFI and MD in predicting driving limitation. The AUC was 0.663 for VFI and 0.650 for MD. The dashed line indicates the reference line (AUC = 0.5). Abbreviations: ROC, receiver operating characteristic; AUC, area under the curve; VFI, Visual Field Index; MD, Mean Deviation.

ROC curves are presented in Figure 1. Given the modest discriminatory performance and limited sensitivity observed in this cohort, these values should be interpreted as exploratory reference points rather than clinically actionable thresholds.

### 3.7. Driving Status and Additional Analyses

When non-drivers were further stratified, significant between-group differences were observed (Kruskal–Wallis test: *p* = 0.0048 for VFI and *p* = 0.0058 for MD). Participants who had ceased driving demonstrated worse visual field indices than active drivers, whereas those who had never driven showed no consistent association with visual field impairment.

Among active drivers, objective visual field indices did not differ significantly between those with and without subjective complaints, suggesting possible behavioural adaptation or selective driving continuation among patients with preserved functional vision.

### 3.8. Inter-Eye Asymmetry

Inter-eye asymmetry showed a non-significant trend toward association with driving limitation, with greater asymmetry observed in functionally impaired participants.

### 3.9. Driving Incidents

Driving incidents were infrequent in the cohort. Although participants reporting incidents tended to have worse visual field indices, differences did not reach statistical significance, likely because of the limited number of events.

### 3.10. Integrated Interpretation of Stratified Analysis

The incorporation of functional severity stratification revealed a consistent gradient linking worsening visual field loss with increased behavioural driving restriction. This graded relationship underscores the clinical relevance of functional visual impairment in real-world activities and highlights behavioural self-regulation as an important adaptive response in patients with progressive glaucomatous damage.

## 4. Discussion

The present study demonstrates a significant association between glaucomatous visual field loss and self-reported driving restriction, rather than objectively measured driving performance or traffic safety outcomes. Worsening visual field status was accompanied by progressively greater restriction of driving activity, including reduced driving frequency, avoidance of night driving, and driving cessation. These findings are consistent with previous studies reporting reduced mobility and driving participation in patients with glaucoma [4,5,6].

The relatively weak concordance between subjective driving complaints and objective visual field indices suggests that self-perceived functional ability should not be considered a reliable surrogate for structural or perimetric impairment. While self-regulation may represent an adaptive behavioural response, objective visual field parameters, particularly VFI and MD in the better eye, may provide useful clinical markers when discussing driving-related functional difficulties. Subjective confidence alone may underestimate functional limitation, especially in patients with preserved central acuity.

From a practical standpoint, patients approaching the identified threshold range (VFI 70–80% or MD −10 to −13 dB) may benefit from closer follow-up and explicit counselling regarding potential driving difficulties. In progressive disease, the interval of reassessment should reasonably reflect the estimated rate of visual field progression, as rapidly progressing patients may cross functional thresholds within a short time frame, whereas stable patients may not require intensified monitoring. Although precise re-evaluation intervals cannot be defined from cross-sectional data, integrating progression rate into follow-up planning may enhance individualized risk assessment.

Across Europe, visual standards for driving are broadly guided by European Union recommendations requiring a horizontal binocular visual field of at least 120° without significant central defects. However, the practical implementation of these standards varies considerably between countries, particularly regarding licence renewal and monitoring of progressive eye disease [14,15].

In countries such as Germany and Finland, medical evaluation, including assessment of visual function, is more consistently integrated into licence renewal procedures, especially in older drivers [16,17]. In contrast, the United Kingdom relies largely on self-reporting of visual conditions and a basic functional acuity check (e.g., number plate recognition), without routine perimetric testing unless a condition is declared [17,18]. Other countries apply intermediate approaches, with medical certification required at certain ages but without systematic visual field reassessment [19,20,21].

In Bulgaria, medical certification for driving licence issuance and renewal is required; however, structured reassessment of visual field function in patients with progressive conditions such as glaucoma is not routinely mandated [22]. In contrast to countries where licence renewal is linked to structured, age-dependent visual reassessment, such as Denmark, Italy, and Spain, Bulgaria does not implement a standardized schedule for repeat ophthalmic evaluation in older drivers or in individuals with progressive disease [19,20,21,22].

Consequently, continued fitness-to-drive decisions often depend on patient self-reporting and the clinical judgement of the examining physician rather than on predefined functional reassessment criteria.

From a practical clinical standpoint, objective visual field parameters may provide an important basis for clinical risk stratification, particularly in patients with moderate or progressive disease. Subjective driving confidence alone appears insufficient, as our data demonstrated limited concordance between perceived difficulty and measurable functional loss. However, subjective self-regulation remains clinically relevant as an early behavioural marker of emerging difficulty. Accordingly, ophthalmologists, general practitioners, and physicians involved in driving-related functional assessment may consider objective perimetric indices, especially MD and VFI in the better eye, while incorporating patient-reported difficulties as supportive, but not decisive, information.

From a clinical perspective, this heterogeneity places ophthalmologists in an important clinical role. In the absence of uniform, disease-specific criteria for glaucoma, decisions regarding driving frequently rely on individual judgement. The functional thresholds identified in the present study may therefore provide practical reference points to support more consistent counselling and documentation, particularly in healthcare systems where formal re-evaluation of visual fields is not systematically embedded in licence renewal procedures.

The identified threshold range (approximately VFI 70–80% and MD −10 to −13 dB) may represent an exploratory transition zone at which self-reported driving difficulties become more likely. However, these values should be interpreted with caution. Given the modest discriminatory performance and limited sensitivity observed in ROC analysis, they should not be considered clinically actionable or definitive licensing criteria. Instead, they may serve as hypothesis-generating reference points for future prospective validation.

An important finding of the study is the distinction between behavioural adaptation and objective driving safety. Many patients appear to self-regulate their driving before complete cessation, particularly by avoiding challenging situations such as night driving. Similar compensatory behavioural patterns have been described in visually impaired older drivers and in glaucoma cohorts [8,9]. However, self-regulation should not be interpreted as equivalent to collision risk or objectively measured driving safety.

The additional analysis of non-drivers further supports this interpretation. Participants who had ceased driving showed significantly worse visual field indices than active drivers, whereas those who had never driven showed no consistent relationship with visual field loss. This suggests that driving cessation is more closely linked to functional impairment than to the mere absence of driving experience.

In addition, decisions to restrict or cease driving may also be influenced by transportation availability, family support, urban environment, and sociocultural factors beyond visual function alone.

The observed association between male sex and a lower likelihood of driving limitation may reflect behavioural or sociocultural differences in driving habits and self-regulation. Previous studies have suggested that men may be less likely to restrict driving despite health-related limitations [11]. This finding should be interpreted cautiously but may be relevant in patient counselling.

The study has several limitations. First, its cross-sectional design precludes conclusions regarding temporal or causal relationships. Second, driving behaviour was assessed using a study-specific self-reported questionnaire that was not formally validated and may be influenced by recall bias, reporting bias, and social desirability bias. Participants may have underreported or overreported driving difficulties, behavioural restrictions, or compensatory self-regulation, which could affect the observed associations. Third, no objective measures of driving performance, such as simulator-based or on-road assessment, were available; such methods may provide complementary insight into real-world driving behaviour [6]. Fourth, the study was conducted in a single tertiary centre, which may limit generalizability to other healthcare systems or populations. Finally, MD and VFI are global indices and do not capture the spatial location of visual field defects, while the sample size may have limited power for detecting more subtle associations, such as inter-eye asymmetry.

Future prospective multicentre studies incorporating binocular visual field measures and objective driving assessments are needed to validate these findings and refine clinically relevant reference ranges for functional driving impairment in glaucoma.

## 5. Conclusions

Glaucomatous visual field loss was significantly associated with self-reported driving limitation and behavioural adaptation of driving habits. Progressive deterioration in visual field status was accompanied by a greater likelihood of reduced driving frequency, avoidance of challenging driving conditions, and driving cessation.

Objective perimetric parameters, particularly VFI and MD, may provide useful contextual information when discussing potential driving difficulties with patients. Threshold values identified in this study should be interpreted as exploratory reference points associated with self-reported driving restriction and require further validation before clinical application.

## Figures and Tables

**Table 1:** Clinical and functional characteristics according to functional severity stratification.

Variable	Green (Preserved)	Yellow (Borderline)	Red (Definite Impairment)	*p*-Value (Trend)
*N*	54	21	25	
Age (years), mean ± SD	62.4 ± 14.9	65.8 ± 13.6	66.9 ± 13.1	0.18
Male, *n* (%)	26 (48%)	11 (52%)	15 (60%)	
Cataract present, *n* (%)	59%	76%	84%	0.04
Comorbidities, *n* (%)	67%	86%	92%	0.02
VFI (better eye), median (IQR)	97 (90–99)	84 (68–94)	62 (45–78)	<0.001
MD (dB), median (IQR)	−2.9 (−5.8 to −0.9)	−7.6 (−12.1 to −3.2)	−14.8 (−20.5 to −8.4)	<0.001
PSD, median (IQR)	2.5	4.8	6.1	0.06
Driving limitation, *n* (%)	17%	48%	72%	<0.001
Non-drivers, *n* (%)	9%	24%	28%	<0.001
Subjective driving complaints, *n* (%)	28%	52%	68%	0.01

Participants were stratified into three groups based on predefined clinical interpretation of visual field data: preserved functional vision, borderline or suspected impairment, and definite glaucomatous functional impairment. Visual field indices demonstrated a progressive deterioration across the three groups, accompanied by increasing prevalence of driving limitation and non-driving status. Trend *p*-values were calculated using non-parametric trend analysis to assess graded relationships between functional severity and clinical outcomes. (SD, standard deviation; IQR, interquartile range; VFI, Visual Field Index; MD, Mean Deviation; PSD, Pattern Standard Deviation; *n*, number of participants).

**Table 2:** Multivariable logistic regression models for predictors of driving limitation.

Variable	Model 1 (VFI) OR (95% CI)	*p*-Value	Model 2 (MD) OR (95% CI)	*p*-Value
VFI (per 1%)	0.972 (0.948–0.996)	0.021	–	–
MD (per 1 dB)	–	–	0.924 (0.875–0.976)	0.004
Male sex	0.20 (0.08–0.52)	0.001	0.21 (0.08–0.53)	0.001
Age	1.01 (0.98–1.04)	0.37	1.01 (0.98–1.04)	0.39
Cataract	1.12 (0.62–2.05)	0.61	1.15 (0.63–2.08)	0.58
Comorbidities	1.18 (0.67–2.33)	0.45	1.20 (0.69–2.36)	0.44

Binary logistic regression was performed to evaluate independent associations between visual field parameters and driving limitation. Model 1 included VFI; Model 2 included MD. Both models were adjusted for age, sex, cataract status, and systemic comorbidities. Abbreviations: OR, odds ratio; CI, confidence interval; VFI, Visual Field Index; MD, Mean Deviation.

## Data Availability

The raw data supporting the conclusions of this article will be made available by the authors on request.

## Abbreviations

The following abbreviations are used in this manuscript

MD	Mean Deviation
VFI	Visual Field Index
PSD	Pattern Standard Deviation
ROC	Receiver Operating Characteristic
AUC	Area Under the Curve
OR	Odds Ratio
CI	Confidence Interval
SD	Standard Deviation
IQR	Interquartile Range
SPSS	Statistical Package for the Social Sciences
